# The Role of Indocyanine Green Angiography and Axillary Reverse Mapping in Breast Reconstruction Surgery

**DOI:** 10.3390/jcm15041638

**Published:** 2026-02-21

**Authors:** Teodora Mihaela Peleaşǎ, Aniela Nodiți-Cuc, Rǎzvan Ioan Andrei, Maria Teodora Popa, Alexandru Blidaru

**Affiliations:** 1 Doctoral School, “Carol Davila” University of Medicine and Pharmacy, 010221 Bucharest, Romania; teodora-mihaela.peleasa@drd.umfcd.ro (T.M.P.); ioan-razvan.andrei@umfcd.ro (R.I.A.); maria-teodora.popa@drd.umfcd.ro (M.T.P.); 2 Surgical Oncology, Department of General Surgery, “Carol Davila” University of Medicine and Pharmacy, 050474 Bucharest, Romania; alexandru.blidaru@umfcd.ro; 3 Department of Surgical Oncology, Institute of Oncology “Prof. Dr. Al. Trestioreanu”, 022328 Bucharest, Romania; 4 Department of Plastic and Reconstructive Surgery, “Prof. Dr. Agrippa Ionescu” Emergency Clinical Hospital, 011356 Bucharest, Romania

**Keywords:** indocyanine green, breast cancer, implant-based breast reconstruction, ischemic complications, mastectomy skin flap necrosis, axillary reverse mapping, lymphedema

## Abstract

**Introduction**: Implant-based breast reconstruction is associated with an increased risk of ischemic complications, which may result in implant loss, suboptimal aesthetic outcomes, and delays in adjuvant oncological treatment. Additionally, axillary surgery carries a risk of upper-limb lymphedema. Indocyanine green (ICG) angiography enables more accurate real-time assessment of tissue perfusion than clinical evaluation alone, while axillary reverse mapping (ARM) facilitates the preservation of upper-limb lymphatics. The integration of these techniques reduces complications and improves both functional and aesthetic outcomes. **Materials and methods**: A total of 208 breast cancer patients who underwent mastectomy followed by immediate implant-based breast reconstruction were enrolled in this case–control study. The prospective intervention group received intraoperative ICG angiography at three time points and underwent ARM with ICG. Conventional surgical techniques were applied in the retrospective control group. **Results**: ICG angiography showed excellent diagnostic accuracy for predicting postoperative ischemic complications (AUC = 0.93, 95% CI 0.82–0.99, *p* < 0.001). Compared with the control group, patients in the ICG group had significantly lower rates of mastectomy skin flap necrosis (11.5% vs. 30.8%, *p* = 0.001), seroma (4.8% vs. 14.4%, *p* = 0.032), hematoma (1.9% vs. 9.6%, *p* = 0.033), and lymphedema (2.9% vs. 17.3%, *p* < 0.001). They also experienced shorter hospitalization (6.2 ± 1.9 vs. 8.0 ± 2.8 days, *p* < 0.001), fewer delays in adjuvant treatment initiation (16.3% vs. 32.7%, *p* = 0.010), and higher aesthetic satisfaction scores (81.41 ± 10.12 vs. 76.03 ± 9.74, *p* <0.001). **Conclusions**: Intraoperative indocyanine green angiography is a valuable tool for predicting ischemic complications in alloplastic breast reconstruction and is associated with reduced morbidity, fewer delays in adjuvant treatment, and improved aesthetic outcomes. Preliminary evidence suggests that axillary reverse mapping is associated with lower rates of upper-limb lymphedema.

## 1. Introduction

Breast cancer represents the leading malignancy diagnosed worldwide, and affects one in eight women during their lifetime [1,2]. While breast-conserving surgery remains the first surgical option in early breast cancer patients, in some cases, mastectomy is still necessary. Axillary staging, conducted by sentinel lymph node biopsy (SLNB) or axillary lymph node dissection (ALND), is an integral component of breast cancer surgery [3].

In patients who require mastectomy, breast reconstruction is associated with major quality of life improvements, but has high complication rates [4]. One of them is represented by mastectomy skin flap necrosis (MSFN), having a reported incidence of 5–30% [5]. The development of MSFN can lead to other postoperative complications, including implant loss, and may require additional surgical interventions. These complications impact the cosmetic and psychological results and may cause delays in postoperative chemotherapy or radiation therapy, affecting long-term oncological outcomes [6]. MSFN can be prevented by an appropriate assessment of the skin flap perfusion. The accurate evaluation through clinical assessment is challenging, and more standardized techniques are required for optimal results [7].

Breast cancer-related lymphedema (BCRL) frequently occurs as a complication following axillary surgery and negatively impacts quality of life by causing discomfort and functional limitation. Disruption of upper-limb lymphatics during sentinel lymph node biopsy and axillary lymph node dissection leads to lymphedema rates of 5% for SLNB and 20–50% for ALND [8,9]. It is considered that the lymphatic drainage of the arm and breast occurs through separate pathways. This hypothesis enables the preservation of arm-draining lymphatics, thereby reducing the risk of lymphedema [10].

Indocyanine green (ICG) was approved for medical purposes in 1956, and since then, its applications have expanded into various medical and surgical specialties [11,12]. In breast cancer surgery, its use has experienced a significant increase over the last decades. The indications include tumor localization, margin assessment, sentinel lymph node identification, axillary reverse mapping, and flap perfusion assessment [13].

ICG angiography (ICGA) allows immediate skin flap perfusion assessment and identification of potential ischemic areas [6,14]. In patients who undergo nipple-sparing mastectomy (NSM), skin-sparing mastectomy (SSM), or skin-reducing mastectomy (SRM) with immediate implant-based breast reconstruction (IBBR), this technique was shown to minimize ischemic complications while enhancing surgical outcomes [15].

Axillary reverse mapping using ICG during axillary surgery preserves upper-limb lymphatic drainage and significantly reduces the incidence of breast cancer-related lymphedema without compromising oncological outcomes [16].

This study investigates the role of intraoperative indocyanine green angiography in guiding surgical decision-making and predicting postoperative ischemia. It also evaluates the impact of ICGA on reducing ischemic complications and improving postoperative outcomes in breast cancer surgery, as well as the effectiveness of axillary reverse mapping in decreasing lymphedema rates.

## 2. Materials and Methods

### 2.1. Study Design

This non-randomized comparative cohort study included a prospective cohort of patients who received ICG angiography and ARM with ICG during their breast cancer surgery from February 2024 to April 2025, as well as a retrospective cohort of patients who did not receive ICG angiography or ARM.

### 2.2. Patient Population

The intervention group consisted of 104 patients who underwent intraoperative ICG angiography and ARM during mastectomy and immediate IBBR surgery. Inclusion criteria were patients over 18 years old, with a surgical indication of mastectomy with immediate IBBR for breast cancer. Exclusion criteria were patients with vascular diseases such as peripheral arterial disease or deep vein thrombosis, patients with known contraindication to ICG according to the protocol (such as iodine or shellfish allergies), and those who could not provide informed consent or follow study requirements.

### 2.3. Intervention

The standard ICGA protocol included three evaluations of skin flap vascularization: E1, E2, and E3.

Patients received 3 mL of indocyanine green solution (25 mg/10 mL; Verdye^®^, Diagnostic Green GmbH, Aschheim-Dornach, Germany) through intravenous injection from a peripheral vein immediately before the breast surgery incision (E1), followed by a 10 mL venous flush of 0.9% Sodium Chloride solution. A near-infrared (NIR) imaging system (IMAGE1 S™ Rubina^®^, KARL STORZ, Tuttlingen, Germany) was used by the surgical team for real-time fluorescence angiography in order to evaluate tissue perfusion and identify vessels to preserve. These vessels were marked and, if possible, preserved during surgery.

A second 3 mL dose of ICG was injected after mastectomy and implant placement (E2). Tissue blood flow evaluation began after the injection, with the first minute crucial for decision-making. The surgical team adjusted the procedure based on intraoperative angiographic findings to optimize blood flow and minimize ischemic complications. Small peri-incisional ischemic areas identified during ICGA were excised. Extended ischemic flaps required the placement of a tissue expander.

A third 3 mL injection of ICG was administered at the end of the surgery (E3), with tissue perfusion again assessed within the first minute. If signs of ischemia persisted, postoperative measures such as topical nitroglycerin application and negative-pressure wound therapy (NPWT) were applied to further minimize complications.

**Figure 1:**
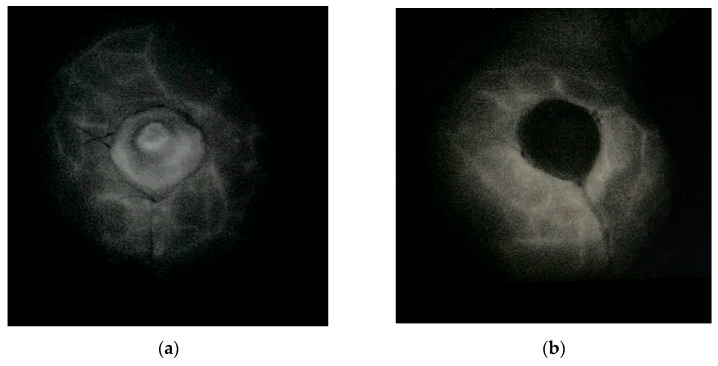
Intraoperative aspects during ICG angiography: (**a**) Normal perfusion; (**b**) Severely decreased perfusion.

During ICGA, at E2 and E3, skin flap perfusion was qualitatively assessed by three surgeons based on color and delay in fluorescence filling, as described in Table 1 (Figure 1).

The ARM procedure involved intradermal injections of 0.4 mL of indocyanine green solution (25 mg/10 mL) into the first and fourth interdigital spaces, as well as the ulnar and radial borders of the wrist. The fluorescence signal was monitored as it traveled from the upper extremity to the axilla for 10 to 15 min using an NIR imaging system. Throughout the axillary surgery, the surgical field was continuously assessed using the NIR camera. Axillary lymph nodes and lymphatics draining the upper extremity were visualized as fluorescent spots and streams.

### 2.4. Control Group

The retrospective control group consisted of 104 patients who underwent mastectomy and immediate implant-based breast reconstruction surgery without ICGA or ARM. Medical records of surgeries performed from January 2022 to January 2024 provided the data for patients included in this group. The patients were matched in terms of age, comorbidities, disease characteristics, neoadjuvant systemic treatment, and surgical procedure type to achieve group comparability.

### 2.5. Surgeries

All surgeries were performed under general anesthesia. In each group, NSM, SSM, or SRM were performed via various skin incisions. Retroareolar intraoperative frozen section was routinely performed for all NSM. Axillary surgery consisted of SLNB or ALND. In the intervention group, all patients also received axillary reverse mapping. After mastectomy, direct-to-implant (DTI) or two-stage (TS) immediate implant-based breast reconstruction was performed.

### 2.6. Complications

**Figure 2:**
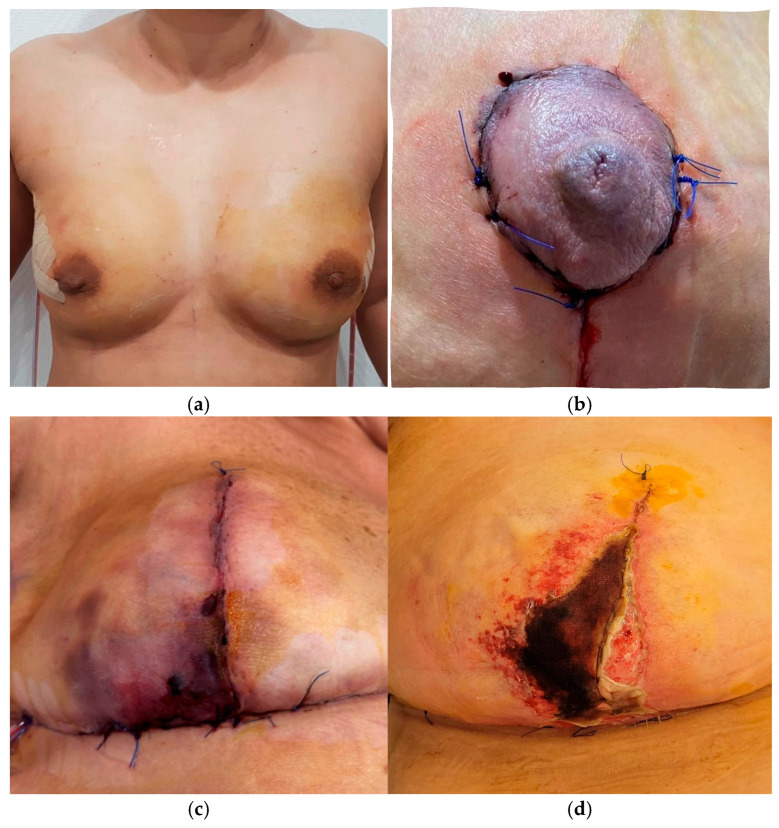
Postoperative aspects: (**a**) Normal aspect; (**b**) Cyanosis; (**c**) Partial-thickness skin flap necrosis; (**d**) Full-thickness skin flap necrosis.

MSFN was evaluated by the SKIN score, which includes a depth score from A to D and a surface area score from 1 to 4. The SKIN depth is evaluated based on the color change of the skin flap, and the surface area score is assigned based on the percentage of breast skin or nipple–areolar complex (NAC) involved [17]. In cases of NSM, the breast mound and NAC are scored separately (Table 2, Figure 2).

### 2.7. Preventive Adjunctive Measures

In both study groups, preventive adjunctive therapies, such as topical nitroglycerin and negative-pressure wound therapy, were administered according to predetermined criteria. In the control group, their use was determined by patient-related risk factors—such as comorbidities, smoking status, and increased body mass index—as well as operative factors, including the extent of dissection, tissue tension, and the surgeon’s intraoperative visual evaluation of tissue perfusion. Decision-making in the intervention group was based on preoperative risk assessment together with intraoperative ICG angiography. Consequently, individuals with high-risk features or ICGA perfusion grades 1–3 received NPWT and/or topical nitroglycerin.

### 2.8. Follow-Up

Follow-up evaluations were performed at 1 week, 2 weeks, 4 weeks, 3 months, and 6 months postoperatively and included overall assessment, clinical examination, lymphedema assessment, and ultrasound. Quality of life (QoL) and satisfaction were evaluated using the BREAST-Q score. Patients completed the Romanian version of the BREAST-Q 1.0 pre-reconstruction module before surgery (at baseline), and the BREAST-Q 1.0 post-reconstruction module at 6-month follow-up.

### 2.9. Definition of Primary Outcomes

The main study objective was to determine:

- Ischemia during ICGA: The number of patients and severity of ischemic areas during intraoperative ICGA, described as grades of ischemia.
- Mastectomy Skin Flap Necrosis: The number of patients, severity, and extent of MFSN, described based on the SKIN score.
- Lymphedema: The number of patients with breast cancer-related lymphedema.

### 2.10. Definition of Secondary Outcomes

The research incorporates secondary results that consist of:

- Complications: The number of patients who needed medical or surgical intervention to address wound dehiscence, infection, seroma, hematoma, and implant loss.
- Need for Additional Surgical Interventions: The number of patients requiring additional surgeries due to complications.
- Length of Hospital Stay: The hospitalization time included all days between the surgical day and the release date.
- Time to Adjuvant Treatment: The time from the date of surgery to the initiation of adjuvant treatment.
- Overall Satisfaction with Aesthetic Outcomes: The patient-reported scores evaluating the postoperative satisfaction with breasts.

### 2.11. Data Collection

Data from the ICG angiography group were collected prospectively through standardized clinical evaluations, operative reports, and scheduled postoperative follow-up visits as per the STROBE guidelines. Medical records provided data on the control group through their surgical notes, which recorded complications and follow-up evaluations.

### 2.12. Variable

Preoperative variables included age, weight, height, comorbidities, smoking status, tumor biology, genetic mutation status, and neoadjuvant systemic treatment (NST). Intraoperative data included type of mastectomy, incision length, implant characteristics, perfusion at E1, E2, and E3, and surgeon’s decision at each point based on these findings. Postoperative data included hospitalization time, complications, histopathological findings, time to adjuvant treatment initiation, adjuvant therapies, lymphedema status, and aesthetic satisfaction auto-evaluation.

### 2.13. Statistical Analysis

Statistical analysis was performed using IBM SPSS Statistics version 31.0.1.0. Continuous variables were presented as mean values and standard deviations (±SD). Dichotomous and categorical data were expressed as frequencies and percentages. An independent *t*-test analysis was utilized to compare continuous variables. For categorical variables, Chi-square and Fisher’s exact tests were used. Logistic regression analysis was performed to identify independent predictors of postoperative ischemic complications. Individual variables were first assessed using univariable analysis, and those with a *p*-value ≤ 0.10 were subsequently entered into the multivariable model. Covariates included use of indocyanine green angiography, incision length, age, smoking status, body mass index, and diabetes mellitus, selected based on clinical relevance, previously reported associations, and results of univariable analyses. To minimize the risk of overfitting, given the limited number of events, the number of variables included in the model was restricted. All statistical tests were two-sided, and statistical significance was defined as a *p*-value < 0.05.

## 3. Results

### 3.1. Patient Demographics and Risk Factors

This research included 208 patients between 27 and 68 years old who received mastectomy with immediate implant-based reconstruction. The intervention group (n = 104) received ICG fluorescence angiography perfusion assessment and axillary reverse mapping during surgery, while the control group (n = 104) did not. The two groups showed similar baseline characteristics, including age, smoking status, diabetes, body mass index (BMI), and neoadjuvant systemic treatment. The patient populations in both groups contained mostly patients with stage I or II disease. The immunohistochemical profiles showed similar distribution between groups (Table 3).

Regarding surgical interventions, the two groups were comparable. Most patients underwent NSM (74.0% versus 71.2%), followed by SSM and SRM. Incision types were “lazy S”, inframammary fold, inverted T, hemibatwing, and elliptic incisions. Mean incision length was also similar. Reconstruction type, plane, and implant volumes were comparable. SLNB was performed in most cases. ARM was performed concomitant with axillary surgery in all patients from the interventional group (Table 4).

### 3.2. Primary Outcomes

#### 3.2.1. Ischemia and MSF

During intraoperative ICGA for skin flap perfusion assessment, 23 patients (22.11%) presented ischemic areas at E2. Out of these, 10 required excision of peri-incisional tissues and 3 required conversion from DTI to two-stage reconstruction.

At the E3 evaluation, 15 patients presented ischemic areas. Postoperatively, 12 women presented ischemic events, and 11 (91.6%) of these events were detected in the E3 time ICGA.

ICGA has high sensitivity (91.7%) and good specificity (85.9%), with excellent negative predictive value (98.9%), but lower positive predictive value (45.8%). The positive likelihood ratio of 6.5 indicates that intraoperative hypoperfusion observed during ICGA increases the probability of necrosis more than six times, while the negative likelihood ratio of 0.10 indicates that a negative result effectively excludes ischemic complications.

When considering the proposed grading system for qualitative evaluation on skin flap perfusion, in only one case (1.25%) of the 80 with grade 0 developed MSFN. Among the 8 patients with grade 1, in one case (12.5%) ischemic complications were noted. The incidence of ischemic complications increased for patients with grade 2 or 3 ICGA findings, with 40% (4 cases) for patients with grade 2 and 100% (6 cases) for those with grade 3.

High risk for complications was considered for patients with high ICGA grades. Grades 0–1 predicted flap viability with a 100% negative predictive value. The cutoff of ≥2 yielded the best balance between sensitivity and specificity (91.7% and 93.5%, respectively) with a Youden Index of 0.85, indicating optimal diagnostic accuracy (Table 5).

**Figure 3:**
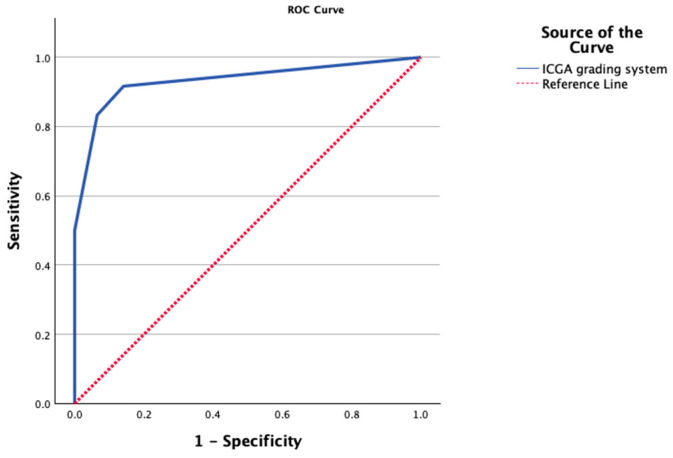
ROC curve for indocyanine green angiography (ICGA) grading system in predicting mastectomy skin flap necrosis.

The ROC analysis demonstrated an excellent discrimination ability of the ICGA grading system for predicting ischemic complications, with an AUC of 0.933 (95% CI: 0.82–0.99, *p* < 0.001) (Figure 3).

In the intervention group, 12 of 104 patients (11.53%) developed MFSN, compared with 32 of 104 patients (30.76%) in the control group. Nine patients from the ICG group required conservative management, including topical application of vasodilator, NPWT, debridement, and hirudotherapy. Three patients underwent surgical reintervention, two for debridement and secondary closure, and one for implant removal. In the control group, 21 cases were conservatively managed, 7 required debridement and closure, and 4 needed implant removal. Mastectomy skin flap necrosis was 2.66 times more frequent in the control group (*p* = 0.04).

To assess the effect of ICGA, patients were grouped based on depth of MSFN as observed in Table 6. Patients in the intervention group had significantly reduced ischemia, superficial necrosis, and deep necrosis rates (6.7% vs. 16.3%, *p* = 0.049; 2.9% vs. 8.7%, *p* = 0.041; and 1.9% vs. 5.8%, *p* = 0.041, respectively).

#### 3.2.2. Lymphedema

Lymphedema occurred in 3 patients (2.9%) in the ICG group, in which ARM was systematically performed, compared with 18 patients (17.3%) in the control group. ARM was associated with a significantly lower risk of lymphedema (RR = 0.14, 95% CI 0.04–0.50, *p* ≤ 0.001), corresponding to an absolute risk reduction of 14.4%, a relative risk reduction of 83.3%, and a number needed to treat of 6.9.

### 3.3. Predictors for Ischemic Complications

On univariate analysis, several patient- and surgery-related factors were associated with postoperative complications. Smoking, diabetes, and obesity (BMI > 30) were significantly correlated with higher ischemic complication rates (52.3% vs. 12.8%, *p* < 0.001; 43.8% vs. 14.4%, *p* < 0.001; and 52.4% vs. 13.3%, *p* < 0.001, respectively). Conversely, intraoperative ICG angiography use was associated with a substantially lower incidence of ischemic events (11.5% vs. 30.8%, *p* = 0.001).

ICGA significantly reduced complication risk across most subgroups (smokers, non-smokers, non-diabetics, BMI ≤ 30). Diabetic and obese patients still benefited, but the differences did not reach statistical significance.

The strongest protective effect was seen in non-obese, non-smoking, and non-diabetic patients, with a relative risk reduction of over 70%. The number needed to treat ranged from 2.4 among smokers to 6.6 among non-smokers (Table 7).

In a logistic regression model, ICG angiography remained a strong independent protective factor, reducing the risk of ischemic complications with 90% (OR = 0.10, 95% CI: 0.03–0.32, *p* ≤ 0.001). Each additional centimeter of incision length significantly increased the risk of necrosis (OR = 1.18, 95% CI: 1.02–1.38, *p* = 0.030). Smoking (OR = 22.97, 95% CI: 7.06–74.65, *p* ≤ 0.001), BMI >30 (OR = 8.37, 95% CI: 2.47–28.31, *p* ≤ 0.001), and diabetes (OR = 3.97, 95% CI: 1.22–12.91, *p* = 0.022) were all identified as independent predictors of postoperative ischemic complications. (Table 8).

### 3.4. Secondary Outcomes

#### 3.4.1. Wound Dehiscence

In the ICG group, wound dehiscence occurred in 16 patients (15.4%) compared to 24 patients (23.1%) in the control group. Although the rate was lower in the ICG group, the difference was not statistically significant (*p* = 0.218). Dehiscence was typically managed conservatively, but one patient from the control group required surgical debridement and secondary closure.

#### 3.4.2. Infection

The ICG group recorded 8 postoperative infections, accounting for 7.7% of patients. The patients received either oral or intravenous antibiotic therapy. The control group developed 18 surgical site infections (17.3%), with two patients needing long-term antibiotics and wound care (*p* = 0.058). No systemic infections were observed throughout the study.

#### 3.4.3. Seroma

The ICG group recorded 5 patients (4.8%) who developed seromas. All patients received successful treatment through ultrasound-guided needle aspirations. In the control group, 15 patients (14.4%) developed seromas, with two needing repeated aspirations and rifampicin instillation followed by aspirative drainage placement (*p* = 0.032).

#### 3.4.4. Hematoma

The ICG group experienced 2 postoperative hematomas (1.9%), while in the control group, 10 patients developed hematomas (9.6%) (*p* = 0.033). One patient from the intervention group and 2 from the control group needed reintervention, while the rest were conservatively managed.

#### 3.4.5. Implant Loss

One patient (1.0%) from the ICG group needed to have their implants removed. Wound stabilization preceded the placement of a tissue expander. The ICG group experienced fewer implant losses than the control group since 5 patients (4.8%) in the control group needed delayed surgery due to ischemic complications (*p* = 0.212).

#### 3.4.6. Need for Additional Surgical Interventions

The ICG group needed 4 patients (3.8%) to undergo unplanned surgical procedures, which included minor flap adjustments and major revision surgeries. The control group experienced 18 patients (17.3%) who needed reoperations because of dehiscence and necrosis, together with implant-related issues (*p* = 0.003).

#### 3.4.7. Length of Hospital Stay

The patients in the ICG group spent an average of 6.2 ± 1.9 days (range: 3–9 days) in the hospital, whereas the control group patients stayed for 8.0 ± 2.8 days (range: 4–12 days) (*p* < 0.001).

#### 3.4.8. Time to Adjuvant Treatment

The patients in the ICG group initiated adjuvant systemic treatments more promptly than the control group, with an average of 4.9 ± 1.6 weeks compared with 5.4 ± 1.7 weeks in the control group (*p* = 0.021). Fewer patients in the ICG group experienced delays beyond 6 weeks compared to controls (16.3% vs. 32.7%, *p* = 0.010). The prolonged healing time because of postoperative wound complications caused most of the delays in the control group.

#### 3.4.9. Aesthetic Outcomes

The Breast-Q questionnaire showed that patients in the ICG group achieved higher postoperative satisfaction with breast scores (mean: 81.41 ± 10.12/100) than those in the control group (mean: 76.03 ± 9.74/100). The mean difference of 5.39 ± 1.38 (95% CI 2.67–8.10, *p* < 0.001) corresponded to a Cohen’s d of 0.54, indicating a moderate effect. Both groups showed an improvement from preoperative to postoperative scores. In the ICGA group, scores increased from 73.14 ± 10.99 to 81.41 ± 10.12 (mean difference 8.27 ± 4.37, 95% CI 7.42–9.12, *p* < 0.001), while in the control group, from 70.92 ± 9.79 to 76.03 ± 9.74 (mean difference 5.11 ± 3.52, 95% CI 4.42–5.79, *p* < 0.001). The improvement was significantly greater in the ICG group, with a mean difference of 3.17 ± 0.55 (95% CI 2.08–4.25, *p* < 0.001), corresponding to a Cohen’s d of 0.79, indicating a moderate-to-large effect.

ICGA showed a positive effect on all secondary outcomes, but the reduction in wound dehiscence, infection, and implant loss rates did not reach statistical significance (Table 9).

## 4. Discussion

Although breast-conserving surgery is the first option in early-stage breast cancer, sometimes mastectomy is mandatory. In these cases, immediate breast reconstruction must be considered, as it improves patients’ quality of life [18,19].

Not surprisingly, the majority of patients received NSM, 74% in the interventional group and 71.2% in the control group. If IBBR is possible, several types of conservative mastectomies can be performed. Both NSM and SSM have similar rates of local recurrence, disease-free survival, and overall survival [20]. SRM is necessary in patients with large and ptotic breasts to achieve acceptable results. In this case, depending on the distance from the sternal notch to the areola, the NAC can be preserved on one or more vascular pedicles or grafted in the desired position [21,22]. In all of these cases, NAC preservation is associated with higher patient-reported satisfaction [22,23]. If there are no signs of NAC involvement and NSM is feasible, NAC preservation is preferred [18].

Among incision types, the lateral IMF incision was preferred in NSM, and the wise-pattern for SRM. Appropriate incision location is important to achieve oncological safety and the desired aesthetic result. For NSM, periareolar incisions have the highest rates of ischemic complications, and also increase the risk of sensory loss [24]. An inframammary fold incision, a radial, or a lateral one may be preferred, as they are associated with lower ischemic risk [25]. The laterally displaced IMF approach is recommended, as it preserves the anterior intercostal artery perforator [26]. For SRM, a wise pattern incision is preferred, even if it is associated with higher ischemic complications than the vertical pattern [27].

In the current study, all patients underwent immediate implant-based breast reconstruction. In the majority of cases (59.6% in the interventional group and 55.7% in the control group), DTI reconstruction was performed, with the prepectoral pocket preferred (59% and 49%, respectively). Both immediate and delayed breast reconstructions are oncologically safe and have similar long-term QoL and satisfaction [28,29]. However, in patients who require post-mastectomy radiotherapy, Immediate IBBR is associated with fewer reinterventions than delayed reconstructions [30]. Immediate breast reconstruction is preferred to delayed breast reconstruction, as it may reduce the period of psychological distress [31]. In low-risk patients with good subcutaneous tissue, DTI is preferred. Otherwise, a two-stage approach may be necessary [32,33]. In both cases, the silicone implant or the tissue expander can be placed subpectoral or prepectoral. DTI breast reconstruction using a prepectoral approach is associated with lower complications, reoperations, and better psychosocial, sexual, and physical well-being scores [34]. Prepectoral tissue expander placement is associated with a similar risk of complications, but with reduced pain, improved aesthetic result, and improved QoL [35,36].

When considering axillary surgery, over 75% of patients in our study underwent sentinel lymph node biopsy. In the context of more neoadjuvant systemic therapy indications, more patients undergo SLNB. ALND is less and less required, even in selected cases with positive lymph nodes [37].

Conservative mastectomy followed by IBBR is associated with high ischemic complication rates [38]. The use of intraoperative ICGA allows surgeons to evaluate tissue perfusion in real time and to change surgical plans [39,40].

We employed a serial ICGA protocol that required 3 evaluations, before surgery, after mastectomy and implant placement, and at the end of the surgery. A 3 mL intravenous injection of ICG was administered, and using a NIR camera, the skin flap vascularization was evaluated in the first 60–90 s. There is no standardized protocol for ICGA during mastectomy and IBBR. Several studies report serial assessments. The optimal time from injection to assessment is considered to be at 60 and 90 s [41].

As expected, this study shows that intraoperative ICGA allows for superior assessment of mastectomy skin flap perfusion, identification and correction of hypoperfused areas, and proper prediction of ischemic complications. The E1 assessment enabled detection of the perforators and adjustments in the incision placement. At E2, a qualitative assessment of skin flap vascularization was performed, and in cases with ischemic areas, re-excision was performed under ICGA guidance. Literature reports that excision of subclinical hypoperfused areas is associated with a significant reduction in ischemic complications and reoperation rates [42].

At E3, another qualitative assessment of skin flap vascularization was performed. A grading system from 0 to 3 was established to describe the degree of skin flap ischemia and demonstrated excellent diagnostic accuracy, with a high sensitivity (91.7%), and specificity (93.5%) for predicting postoperative necrosis. Grades 2–3 were strongly associated with MFSN, while grades 0–1 reliably predicted viable skin flaps with a 100% negative predictive value. Grading was independently confirmed by two additional surgeons, thereby enhancing interobserver reliability and indicating that this qualitative method is reproducible and not operator-dependent. While this qualitative ICG angiography technique showed excellent diagnostic accuracy, it lacks the objective assessment of a quantitative measurement. Studies indicate that combining quantitative analysis with qualitative ICG measurements improves precision and surgical outcomes. Several variables were found to predict ischemic complications. One study found that the interval of time between ICG injection and perfusion of the least vascularized area was the only variable with statistical significance for predicting perfusion levels. The researchers established a cut-off of ≥170 s, which was 100% sensitive for predicting skin flap necrosis [43]. Another study found that patients with 70.5 s or longer from ICG injection to perfusion of the entire skin flap had a significantly higher risk of MSFN [44]. As for cut-off values defining hypoperfused areas, one study found that perfusion below 30% in areas excluding the nipple is an accurate predictor for MSFN [45]. Another study defined it as being under 33% at the end of the reconstruction and showed a significantly higher risk of complications [40].

Identifying high-risk patients at E3 assessment enabled immediate preventive measures, such as topical vasodilator applications and NWPT. The topical application of nitroglycerin and dimethylsulfoxide has been reported to significantly lower the incidence of ischemic complications [46]. The use of NWPT after immediate breast reconstruction is associated with a significant reduction in major complications [47]. Compared with standard dressing, single use of NWPT after tissue expander-to-implant exchanges was also found to improve scar elasticity and aesthetic appearance [40].

Our findings also confirm the value of ICGA in reducing postoperative ischemic complications. The mastectomy skin flap necrosis rate in the intervention group was 11.5%, compared with 30.8% in the control group. The literature reports variable rates of 7% to 30% [5,48,49].

Patients in the ICGA group showed a higher proportion of viable skin flaps (A1 scores) and a markedly lower incidence of ischemia (B2-4), superficial necrosis (C2-4), and deep necrosis (D2-4) compared to the control group. The SKIN score is a scoring system used to describe the severity of MSFN. It is composed of 2 values: one to describe the depth and the other to describe the area of ischemia complications after conservative mastectomy and IBBR. Its purpose is to standardize the assessment of skin necrosis, and it is strongly correlated with the need for reoperations [17].

Studies that employ ICG angiography show lower rates of mastectomy skin flap necrosis, with a recent study finding rates as low as 3.1–8% [20,41,50]. Our research found significantly lower MSFN rates in the ICG group than in the control group. The ICG group experienced significantly reduced skin flap necrosis (11.5 versus 30.8%), leading to a 62.5% relative risk reduction. These findings are similar to the existing literature. A meta-analysis of 26 studies demonstrated that ICG-guided procedures lowered major complications and flap necrosis risk by 44% (OR 0.56, 95% CI 0.42–0.76, *p* = 0.0001) [51].

In both groups, ischemia rates were affected by risk factors, including smoking, diabetes, and BMI, while patients undergoing ICGA had lower rates compared to the control group patients. These findings echo the existing literature, as smoking, diabetes, and obesity are known risk factors that can compromise skin flap perfusion [52,53].

In univariate analysis, the most important risk factor for MSFN was smoking. Higher rates of ischemic complications were found in smokers than in non-smokers (52.3% vs. 12.8%, *p* < 0.001). Even if ICG use is associated with a significant reduction in postoperative complications in both smokers and non-smokers, smoking cessation at least 6 weeks before surgery is an important preventative measure [54].

Ischemia rates for diabetic patients were 43.8% compared to 14.4% in non-diabetic patients (*p* < 0.001). ICG angiography reduced MSFN rates in both diabetic and non-diabetic patients, but for non-diabetic patients, it did not reach statistical significance. However, preoperative control of glycemic levels is recommended in patients undergoing mastectomy and IBBR [54].

Patients with obesity had a higher incidence of ischemic complications (52.4% vs. 13.3%, *p* < 0.001). Intraoperative ICG angiography had a positive effect in patients with normal weight or overweight (BMI ≤ 30), while the benefit in obese patients was present but not significant [54].

In multivariate analysis, ICG angiography was an independent protective factor for MSFN (OR = 0.10, 95% CI 0.03–0.32, *p* < 0.001). Smoking, diabetes, obesity, and increased incision length were independent risk factors, as reported in the literature [5,49,50]. Considering incision length during preoperative planning is an important step. In our study, each additional centimeter increased the risk of complications by 18%. These findings are in line with a recent study that found a 23% increase in risk per additional centimeter [40]. After adjustment for confounder factors, ICGA remained a protective factor for ischemic complications.

Intraoperative ICGA was also associated with better outcomes across several secondary endpoints. In patients who received ICG angiography, other complication rates were significantly lower. ICGA decreased the incidence of seroma and hematoma (4.8% vs. 14.4%, *p* = 0.032; and 1.9% vs. 9.6%, *p* = 0.033, respectively). Several studies reported lower hematoma and seroma rates in the ICG group compared with clinical assessment [55].

Implant loss rates were lower in patients who underwent ICGA. Only 1.0% of patients in the intervention group experienced implant loss, compared to 4.8% in the control group (*p* = 0.212). Although this difference did not reach statistical significance, likely due to the low number of events, these results are consistent with previous reports that demonstrated a significantly reduced risk of overall reconstruction loss when ICG angiography was employed [51].

In the intervention group, wound dehiscence and infection rates were lower, but this difference was not statistically significant. These results are aligned with the existing literature, that report no decrease in minor complications [56].

The hospital stay was also significantly shorter in the ICGA group (6.2 vs. 8.0 days), similar to the existing data [57].

Additionally, intraoperative ICG angiography significantly lowered the need for additional interventions. Only 3.8% of patients in the ICGA group required reinterventions, compared with 17.3% in the control group (*p* = 0.003). The results align with the literature, which shows a significant reduction in reoperation rates with the use of qualitative ICG angiography (RR 0.50, 95% CI: 0.35–0.72) [55].

ICGA significantly reduces the risk of delaying adjuvant treatment initiation. In the intervention group, the interval from surgery to the start of adjuvant therapies was shorter, and fewer patients experienced a delay of over 6 weeks (16.3% vs. 32.7%, *p* < 0.001). ICGA is associated with a lower risk of major postoperative complications, the main reason for a possible delay in postoperative oncological treatment [58,59].

Postoperative satisfaction with breasts was significantly higher in the ICGA group (81.41/100 versus 76.03/100). Aesthetic satisfaction is an important determinant of psychological well-being, self-image, and overall quality of life following breast reconstruction [60]. ICGA reduces the incidence of major complications, including implant loss, thereby improving aesthetic results. The BREAST-Q questionnaire is a validated tool for assessing quality of life [61]. While no studies regarding implant-based breast reconstruction directly report on the effect of ICGA on BREAST-Q scores, several articles found an increase in postoperative scores after ICGA during autologous breast reconstruction [62,63].

In our study, the incidence of lymphedema was significantly lower in the ICG angiography group, in which axillary reverse mapping was systematically performed during axillary surgery (2.9% vs. 17.3%). Breast cancer-related lymphedema is a chronic and debilitating complication after axillary surgery, with a reported incidence of from 5% after SLNB up to 20–50% after ALND [8,9]. By preserving lymphatic vessels that drain the upper arm, ARM reduces the risk of upper arm lymphedema and is associated with better QoL [16].

The primary objective of ICG angiography is to provide an accurate method for assessing skin flap perfusion. This enables surgeons to adjust surgical plans, resulting in lower complication rates and better aesthetic outcomes. Moreover, the combined use of axillary reverse mapping may enhance patients’ quality of life by further reducing the incidence of upper arm lymphedema.

Several limitations should be considered in interpreting these findings. The single-center design and retrospective control group may introduce selection and performance biases, including potential Hawthorne effects and the impact of ongoing improvements in surgical practice. The same core surgical team performed all of the procedures, and perioperative treatment methods were consistent throughout the trial, despite the fact that patients were enrolled at various times. However, the observed outcomes might have been impacted by unmeasured contextual factors, process improvement, and progressive institutional experience. Therefore, residual temporal confounding represents an inherent limitation and should be taken into consideration when evaluating the comparative results. Furthermore, the intervention cohort underwent both ICG angiography and axillary reverse mapping, whereas the control group did not receive either procedure. Consequently, this analysis highlights the clinical impact of a combined intraoperative approach, preventing the separation of the individual effects of each modality and therefore preventing causal attribution to either technique individually. Adjunctive measures, including topical nitroglycerin and negative-pressure wound therapy, were applied in both groups according to standardized criteria. However, the earlier detection of compromised perfusion and prompt intervention in the ICGA group may have contributed to the observed outcome differences. The lack of a quantitative, objective measure for intraoperative skin flap perfusion is an additional limitation. Although the proposed ICGA grading system may inform clinical decision-making, it remains largely subjective. Finally, the relatively short follow-up period may underestimate the true incidence of lymphedema and restrict assessment of long-term quality-of-life outcomes. These findings should therefore be considered preliminary, and extended follow-up is required to confirm their durability.

## 5. Conclusions

The application of ICG angiography during conservative mastectomy and IBBR allows for accurate preoperative mapping, guided intraoperative decision making, and high prediction level of ischemic complications. Its use is associated with significantly lower ischemic complication rates. Moreover, it leads to lower rates of seroma and hematoma, shorter hospitalization time, and no significant delay in adjuvant treatment. By reducing major complication rates, ICGA is associated with better aesthetic results and better quality of life. Additionally, axillary reverse mapping during axillary surgery preserves upper-limb lymphatic drainage and, according to preliminary six-month follow-up data, is associated with reduced rates of breast cancer-related lymphedema.

## Figures and Tables

**Table 1:** Ischemia severity grades during ICGA.

Grade	Description
0	Normal perfusion- uniform bright white coloring (normal)
1	Mildly decreased perfusion- slight delay in coloring
2	Moderately decreased perfusion- patchy or delayed filling (>60 s)
3	Severely decreased perfusion- dark area

**Table 2:** SKIN Score for mastectomy skin flap necrosis (MSFN).

**Depth of MFSN**	
Score	Description
A	No evidence of MFSN
B	Color change of skin flap suggesting impaired perfusion or ischemic injury (may be cyanosis or erythema)
C	Partial-thickness skin flap necrosis resulting in at least epidermal sloughing
D	Full-thickness skin flap necrosis involving dermal and subdermal layers
**Surface Area of MFSN**	
Score	Description
1	None—no change in breast skin or nipple–areolar complex (NAC)
2	Breast: change affects 1–10% of breast skin. NAC: change affects 1–10% of the nipple–areolar complex
3	Breast: change affects 11–30% of breast skin. NAC: change affects 11–30% or total nipple involvement
4	Change affects >30% of breast skin or >30% of NAC surface area

**Table 3:** Clinical Characteristics and Treatments in the Indocyanine Green Angiography (ICGA) and Control Groups.

Variable	ICGA (n = 104)	Control Group (n = 104)
Risk Factors		
Active Smokers, n (%)	24 (23.1%)	20 (19.2%)
Diabetes, n (%)	22 (21.1%)	26 (25.0%)
Mean body mass index (kg/m^2^)	27.6 (18.5–35.2)	28.2 (19.0–34.8)
TNM Stage, n (%)		
Stage I	44 (42.3%)	36 (34.6%)
Stage II	44 (42.3%)	52 (50.0%)
Stage III	16 (15.4%)	16 (15.4%)
Stage IV	0	0
Immunohistochemical Type, n (%)		
Luminal A	28 (26.9%)	26 (25.0%)
Luminal B	48 (46.2%)	46 (44.2%)
HER2 Positive	20 (19.2%)	22 (21.2%)
Triple-Negative	8 (7.7%)	10 (9.6%)
Genetic Mutations, n (%)		
BRCA1/2 Mutation	16 (15.4%)	12 (11.5%)
TP53 Mutation	10 (9.6%)	6 (5.8%)
No Detectable Mutations	78 (75.0%)	86 (82.7%)
Neoadjuvant and Adjuvant Treatment, n (%)		
Neoadjuvant Systemic Treatment	47 (45.1%)	45 (43.2%)
Adjuvant Radiotherapy	20 (19.2%)	24 (23.1%)
Adjuvant Hormone Therapy	96 (92.3%)	94 (90.4%)
Adjuvant Chemotherapy	36 (34.6%)	30 (28.8%)

**Table 4:** Surgical Interventions in the ICGA and Control Groups.

Variable	ICGA (n = 104)	Control Group (n = 104)
Type of Mastectomy, n (%)		
Nipple-sparing mastectomy (NSM)	77 (74.0%)	74 (71.2%)
Skin-sparing mastectomy (SSM)	15 (14.4%)	17 (16.3%)
Skin-reducing, nipple-sparing mastectomy	6 (5.8%)	7 (6.7%)
Skin-reducing mastectomy (SRM)	6 (5.8%)	6 (5.8%)
Type of Incision, n (%)		
Lazy S	29 (27.9%)	24 (23.0%)
Lateral Fold	27 (25.9%)	23 (22.1%)
Inframammary Fold	21 (20.0%)	19 (18.3%)
Inverted T	23 (22.1%)	23 (22.1%)
Hemibatwing	5 (4.8%)	4 (3.8%)
Elliptic	9 (8.6%)	11 (10.6%)
Mean Incision Length (cm)	13.1 ± 2.3	13.4 ± 2.1
Type of Reconstruction, n (%)		
DTI (Direct-to-Implant)	62 (59.6%)	58 (55.7%)
TS (Two-Stage)	42 (40.4%)	46 (44.3%)
Reconstruction Plane, n (%)		
Prepectoral Implant	45 (43.3%)	38 (36.5%)
Retropectoral Implant	17 (16.3%)	22 (21.2%)
Retropectoral Expander	30 (28.8%)	32 (30.8%)
Prepectoral Expander	12 (11.5%)	12 (11.5%)
Axillary Surgery, n (%)		
Sentinel Lymph Node Biopsy (SLNB)	80 (76.9%)	78 (75.0%)
Axillary Lymph Node Dissection (ALND)	24 (23.1%)	26 (25.0%)
Axillary Reverse Mapping (ARM)	Performed in all patients	Not performed

**Table 5:** Diagnostic Performance of ICGA Grades for Predicting Mastectomy Flap Necrosis.

Cutoff (≥Grade)	Sensitivity (%)	Specificity (%)	Positive Predictive Value (%)	Negative Predictive Value (%)	Youden Index (J)
≥1	100.0 (12/12)	85.9 (79/92)	48.9	100.0	0.86
≥2	91.7 (11/12)	93.5 (86/92)	64.7	98.8	0.85
≥3	50.0 (6/12)	100.0 (92/92)	100.0	95.8	0.50

**Table 6:** Surgical Interventions in ICG Angiography and Control Groups.

Score	ICG-A (n = 104)	Control Group (n = 104)	*p*-Value
A1	92 (88.5%)	72 (69.2%)	0.001
B2-4	7 (6.7%)	17 (16.3%)	0.049
C2-4	3 (2.9%)	9 (8.7%)	0.041
D2-4	2 (1.9%)	6 (5.8%)	0.041

**Table 7:** Univariate Analysis of Risk Factors Associated with Postoperative Ischemic Complications.

Risk Factor	Group	Complications n/N (%)	Relative Risk	95% CI	ARR (%)	RRR (%)	NNT	*p*-Value
Smoking	ICG	8/24 (33.3%)	0.17	0.04–0.62	41.7	54.6	2.4	<0.001
	Control	15/20 (75.0%)	—	—	—	—	—	—
Non- Smoking	ICG	4/80 (5.0%)	0.2	0.07–0.65	15.2	75.2	6.6	0.004
	Control	17/84 (20.2%)	—	—	—	—	—	—
Diabetes	ICG	7/22 (31.8%)	0.4	0.12–1.3	22.0	40.9	4.5	0.15
	Control	14/26 (53.8%)	—	—	—	—	—	—
Non- Diabetes	ICG	5/82 (6.1%)	0.22	0.08–0.62	17.0	73.6	5.9	0.003
	Control	18/78 (23.1%)	—	—	—	—	—	—
BMI > 30	ICG	8/20 (40.0%)	0.38	0.11–1.33	23.6	37.1	4.2	0.22
	Control	14/22 (63.6%)	—	—	—	—	—	—
BMI ≤ 30	ICG	4/84 (4.8%)	0.18	0.06–0.55	17.2	78.2	5.8	0.001
	Control	18/82 (22.0%)	—	—	—	—	—	—

**Table 8:** Multivariate Analysis of Risk Factors Associated with Postoperative Ischemic Complications.

Variable	Odds Ratio	95% CI	*p*-Value
ICG Angiography Use	0.10	0.03–0.32	<0.001
Incision Length (per cm increase)	1.18	1.02–1.38	0.030
Diabetes (Yes)	3.97	1.22–12.91	0.022
BMI > 30	8.37	2.47–28.31	<0.001
Smoking (Yes)	22.97	7.06–74.65	<0.001

**Table 9:** Primary and Secondary Outcomes in ICG Group and Control Group.

Outcome	ICG Group (n = 104)	Control Group (n = 104)	RR (95% CI)	*p*-Value
MSFN	12 (11.5%)	32 (30.8%)	0.29 (0.14–0.61)	0.001
Dehiscence	16 (15.4%)	24 (23.1%)	0.61 (0.30–1.22)	0.218
Infection	8 (7.7%)	18 (17.3%)	0.39 (0.16–0.96)	0.058
Seroma	5 (4.8%)	15 (14.4%)	0.30 (0.11–0.86)	0.032
Hematoma	2 (1.9%)	10 (9.6%)	0.18 (0.04–0.83)	0.033
Implant loss	1 (1.0%)	5 (4.8%)	0.19 (0.02–1.68)	0.212
Reoperations	4 (3.8%)	18 (17.3%)	0.19 (0.06–0.57)	0.003
Hospital Stay (days, mean ± SD)	6.2 ± 1.9	8.0 ± 2.8	—	<0.001
Delay of Adjuvant Therapy	17 (16.3%)	34 (32.7%)	0.40 (0.21–0.78)	0.010
Lymphedema	3 (2.9%)	18 (17.3%)	0.14 (0.04–0.50)	<0.001
Aesthetic Satisfaction (Breast-Q)	81.41 ± 10.12	76.03 ± 9.74	—	<0.001

## Data Availability

Data are available upon reasonable request from the corresponding author.

## Abbreviations

The following abbreviations are used in this manuscript:

ICG	Indocyanine Green
ARM	axillary reverse mapping
SLNB	sentinel lymph node biopsy
ALND	axillary lymph node dissection
MSFN	mastectomy skin flap necrosis
BCRL	Breast cancer-related lymphedema
ICGA	Indocyanine Green Angiography
NSM	Nipple-Sparing Mastectomy
SSM	Skin-Sparing Mastectomy
SRM	Skin-Reducing Mastectomy
IBBR	Implant-based breast reconstruction
NIR	near-infrared
NPWT	Negative-pressure wound therapy
DTI	direct-to-implant
TS	two-stage
NAC	nipple-areola complex
QoL	quality of life
NST	neoadjuvant systemic treatment
BMI	Body Mass Index

## References

[1] Sung H., Ferlay J., Siegel R.L., Laversanne M., Soerjomataram I., Jemal A., Bray F. (2021). Global Cancer Statistics 2020: GLOBOCAN Estimates of Incidence and Mortality Worldwide for 36 Cancers in 185 Countries. CA Cancer J. Clin..

[2] Heer E., Harper A., Escandor N., Sung H., McCormack V., Fidler-Benaoudia M.M. (2020). Global burden and trends in premenopausal and postmenopausal breast cancer: A population-based study. Lancet Glob. Health.

[3] Keelan S., Flanagan M., Hill A.D.K. (2021). Evolving Trends in Surgical Management of Breast Cancer: An Analysis of 30 Years of Practice Changing Papers. Front. Oncol..

[4] Chen W., Lv X., Xu X., Gao X., Wang B. (2018). Meta-analysis for psychological impact of breast reconstruction in patients with breast cancer. Breast Cancer.

[5] Robertson S.A., Jeevaratnam J.A., Agrawal A., Cutress R.I. (2017). Mastectomy skin flap necrosis: Challenges and solutions. Breast Cancer.

[6] Nguyen C.L., Dayaratna N., Graham S., Azimi F., Mak C., Pulitano C., Warrier S. (2024). Evolution of Indocyanine Green Fluorescence in Breast and Axilla Surgery: An Australasian Experience. Life.

[7] Frey J.D., Salibian A.A., Choi M., Karp N.S. (2019). The Importance of Tissue Perfusion in Reconstructive Breast Surgery. Plast. Reconstr. Surg..

[8] Sharifi N., Ahmad S. (2024). Breast cancer-related lymphedema: A critical review on recent progress. Surg Oncol..

[9] Slavu I.M., Tulin A., Filipoiu F., Dogaru A., Munteanu O., Anca Monica O.M., Tulin R., Ursut B. (2024). Axillary Lymphadenectomy: Safe Dissection Through a Correct Technique. Cureus.

[10] Abbas Y., Hamdy O. (2023). Axillary reverse mapping in breast cancer: An overview. Breast Dis..

[11] Coufal O., Fait V. (2016). Use of indocyanine green and the HyperEye system for detecting sentinel lymph nodes in breast cancer within a population of European patients: A pilot study. World J. Surg. Oncol..

[12] Alander J.T., Kaartinen I., Laakso A., Patila T., Spillmann T., Tuchin V.V., Venermo M., Välisuo P. (2012). A review of indocyanine green fluorescent imaging in surgery. Int. J. Biomed. Imaging.

[13] Zelken J.A., Tufaro A.P. (2015). Current Trends and Emerging Future of Indocyanine Green Usage in Surgery and Oncology: An Update. Ann. Surg. Oncol..

[14] Oradan A.V., Georgescu A.V., Ilie-Ene A., Corpodean A.A., Juncan T.P., Muntean M.V. (2024). Mastectomy Skin Flap Perfusion Assessment Prior to Breast Reconstruction: A Narrative Review. J. Pers. Med..

[15] Fadell N., Laurent F., Sanka S.A., Ochoa E., Yaeger L., Li X., Wood M.D., Sacks J.M., Bardran S. (2024). The Utility of Indocyanine Green Angiography in Breast Reconstruction to Detect Mastectomy Skin Flap Necrosis and Free Flap Perfusion: An Umbrella Review. Bioengineering.

[16] Fan Y.C., Li L., Meng X.C. (2025). Quality of life and oncologic safety of axillary reverse mapping in patients with breast cancer: A systematic review and meta-analysis. Ann. Med. Surg..

[17] Lemaine V., Hoskin T.L., Farley D.R., Grant C.S., Boughey J.C., Torstenson T.A., Jacobson S.R., Jakub J.W., Degnim A.C. (2015). Introducing the SKIN score: A validated scoring system to assess severity of mastectomy skin flap necrosis. Ann. Surg. Oncol..

[18] Zhong T., Fletcher G.G., Brackstone M., Frank S.G., Hanrahan R., Miragias V., Stevens C., Vesprini D., Vito A., Wright F.C. (2025). Postmastectomy Breast Reconstruction in Patients with Non-Metastatic Breast Cancer: A Systematic Review. Curr. Oncol..

[19] Lee Y.Y., Lai H.W., Guevara A.M., Maldonado J.T., Lin H.Y., Feng C.J., Hwang B.F., Lin S.L., Huang H.I., Siao F.C. (2025). Mastectomy Alone or with Immediate Breast Reconstruction: Trend, Precipitating Factors, Patients Reported Outcome, and Oncologic Safety Analysis with and without Propensity Score Matching from 3759 Mastectomy Patients. Aesthetic Plast. Surg..

[20] Agha R.A., Al Omran Y., Wellstead G., Sagoo H., Barai I., Rajmohan S., Borrelli M.R., Vella-Baldacchino M., Orgill D.P., Rusby J.E. (2019). Systematic review of therapeutic nipple-sparing versus skin-sparing mastectomy. BJS Open.

[21] Lisa A.V.E., Mela A., Miranda S., Alessandri Bonetti M., Bottoni M., Intra M., Pagan E., Bagnardi V., Rietjens M. (2024). Comparative Efficacy of Classic Versus Horizontal Incision Techniques in Skin-Reducing Mastectomy: A Single Center Retrospective Analysis. J. Clin. Med..

[22] Graziano F.D., Levy J., Kim M., Massand S., Shammas R.L., Boe L., Mehrara B.J., Matros E., Nelson J.A., Stern C.S. (2025). Impact of Nipple-Areolar Complex Reconstruction on Patient Reported Outcomes After Alloplastic Breast Reconstruction: A BREAST-Q Analysis. Plast. Reconstr. Surg..

[23] Tulin A.D., Ion D.E., Avino A., Gheoca-Mutu D.E., Abu-Baker A., Tigaran A.E., Timofan T., Ostafi I., Jecan R.C., Raducu L. (2025). Video-Assisted Mastectomy with Immediate Breast Reconstruction: First Clinical Experience and Outcomes in an Eastern European Medical Center. Cancers.

[24] Park S., Yoon C., Bae S.J., Cha C., Kim D., Lee J., Ahn S.G., Roh T.S., Kim Y.S., Jeong J. (2020). Comparison of complications according to incision types in nipple-sparing mastectomy and immediate reconstruction. Breast.

[25] Frey J.D., Salibian A.A., Levine J.P., Karp N.S., Choi M. (2018). Incision Choices in Nipple-Sparing Mastectomy: A Comparative Analysis of Outcomes and Evolution of a Clinical Algorithm. Plast. Reconstr. Surg..

[26] Rancati A.O., Nahabedian M.Y., Angrigiani C., Irigo M., Dorr J., Acquaviva J., Rancati A. (2023). Revascularization of the Nipple-Areola Complex following Nipple-Sparing Mastectomy. Plast. Reconstr. Surg..

[27] Cheong S.C., Maliekkal J., Tung W.S., Saadya A., Awad G.A. (2025). Wise Versus Vertical Mastopexy Pattern Skin-reducing Mastectomy With Immediate Breast Reconstruction: Systematic Review and Meta-analysis. Plast. Reconstr. Surg. Glob. Open..

[28] Gumuscu R., Warnberg F., de Boniface J., Sund M., Ahsberg K., Hansson E., Folkvaljon F., Unukovych D., Mani M. (2024). Timing and type of breast reconstruction in SweBRO 3: Long-term outcomes. Br. J. Surg..

[29] Avino A., Raducu L., Brinduse L.A., Jecan C.R., Lascar I. (2020). Timing between Breast Reconstruction and Oncologic Mastectomy-One Center Experience. Medicina.

[30] Kooijman M.M.L., Hage J.J., Scholten A.N., van Duijnhoven F.H., Breugem C.C., Woerdeman L.A.E. (2025). Advantages of immediate implant-based breast reconstruction over delayed breast reconstruction in women treated with postmastectomy radiotherapy for breast cancer. Breast Cancer Res. Treat..

[31] Roy N., Downes M.H., Ibelli T., Amakiri U.O., Li T., Tebha S.S., Balija T.M., Schnur J.B., Montgomery G.H., Henderson P.W. (2024). The psychological impacts of post-mastectomy breast reconstruction: A systematic review. Ann. Breast Surg..

[32] van der Wielen A., Negenborn V., Burchell G.L., Remmelzwaal S., Lapid O., Driessen C. (2023). Less is more? One-stage versus two-stage implant-based breast reconstruction: A systematic review and meta-analysis of comparative studies. J. Plast. Reconstr. Aesthetetic Surg..

[33] Di Giuli R., Cavallero M.F., Ferrari C., Vaccari S., Bucci F., Bandi V., Klinger F.M., Vinci V. (2025). Two-stage prepectoral breast reconstruction: A comprehensive review and meta-analysis. J. Plast. Reconstr. Aesthetetic Surg..

[34] Cogliandro A., Salzillo R., De Bernardis R., Loria F.S., Petrucci V., Barone M., Tenna S., Cagli B., Persichetti P. (2023). Prepectoral Versus Subpectoral Direct-to-Implant Breast Reconstruction: Evaluation of Patient’s Quality of Life and Satisfaction with BREAST-Q. Aesthetic Plast. Surg..

[35] Escandon J.M., Weiss A., Christiano J.G., Langstein H.N., Escandon L., Prieto P.A., Gooch J.C., Manrique O.J. (2023). Prepectoral versus subpectoral two-stage implant-based breast reconstruction: U.S. medical center experience and narrative review. Ann. Transl. Med..

[36] Vingan P.S., Kim M., Boe L.A., Coriddi M.R., Allen R.J., Disa J.J., Stern C.S., Matros E., Mehrara B.J., Nelson J.A. (2025). One-Year Outcomes in Prepectoral versus Subpectoral Alloplastic Breast Reconstruction. Plast. Reconstr. Surg..

[37] Reimer T., Kuehn T., Mueller V., Ditsch N., Fehm T., Albert U.S., Bartsch R., Bauerfeind I., Bjelic-Radisic V., Blohmer J.U. (2025). AGO Breast Commission recommendations for the surgical therapy of breast cancer: Working Group on Gynecologic Cancers (AGO) update 2025. Eur. J. Surg. Oncol..

[38] Mak J.C., Kwong A. (2020). Complications in Post-mastectomy Immediate Breast Reconstruction: A Ten-year Analysis of Outcomes. Clin. Breast Cancer.

[39] Pruimboom T., van Kuijk S.M.J., Qiu S.S., van den Bos J., Wieringa F.P., van der Hulst R.R.W.J., Schols R.M. (2020). Optimizing Indocyanine Green Fluorescence Angiography in Reconstructive Flap Surgery: A Systematic Review and Ex Vivo Experiments. Surg. Innov..

[40] Cuniolo L., Diaz R., Anastasia D., Murelli F., Cornacchia C., Depaoli F., Gipponi M., Margarino C., Boccardo C., Franchelli S. (2025). Indocyanine Green Angiography to Predict Complications in Subcutaneous Mastectomy: A Single-Center Experience. J. Pers. Med..

[41] Nguyen C.L., Dayaratna N., Easwaralingam N., Seah J.L., Azimi F., Mak C., Pulitano C., Kumar Warrier S. (2025). Developing an Indocyanine Green Angiography Protocol for Predicting Flap Necrosis During Breast Reconstruction. Surg. Innov..

[42] Griffiths M., Chae M.P., Rozen W.M. (2016). Indocyanine green-based fluorescent angiography in breast reconstruction. Gland Surg..

[43] Mastronardi M., Fracon S., Scomersi S., Fezzi M., Bortul M. (2022). Role of Qualitative and Quantitative Indocyanine Green Angiography to Assess Mastectomy Skin Flaps Perfusion in Nipple/Skin-Sparing and Skin-Reducing Mastectomies with Implant-Based Breast Reconstruction. Breast J..

[44] Lee J.K., Jeon B.J., Woo K.J. (2025). Ingress Time as a Metric for Indocyanine Green Angiographic Evaluation of Skin Flap Perfusion in Immediate Implant-Based Reconstruction. Plast. Reconstr. Surg..

[45] Kim J., Han M.W., Hong K.Y. (2024). Prospective Clinical Trial for Predicting Mastectomy Skin Flap Necrosis with Indocyanine Green Angiography in Implant-Based Prepectoral Breast Reconstruction. Aesthetic Plast. Surg..

[46] Tang N., Li H., Chow Y., Blake W. (2023). Non-operative adjuncts for the prevention of mastectomy skin flap necrosis: A systematic review and meta-analysis. ANZ J. Surg..

[47] Akhter H.M., Macdonald C., McCarthy P., Huang Y., Meyer B.R., Shostrum V.K., Cromer K.J., Johnson P.J., Wong S.L., Hon H.H. (2023). Outcomes of Negative Pressure Wound Therapy on Immediate Breast Reconstruction after Mastectomy. Plast. Reconstr. Surg. Glob. Open.

[48] Matsen C.B., Mehrara B., Eaton A., Capko D., Berg A., Stempel M., Van Zee K.J., Pusic A., King T.A., Cody H.S. (2016). Skin Flap Necrosis After Mastectomy With Reconstruction: A Prospective Study. Ann. Surg. Oncol..

[49] Moo T.A., Nelson J.A., Sevilimedu V., Charyn J., Le T.V., Allen R.J., Mehrara B.J., Barrio A.V., Capko D.M., Pilewskie M. (2023). Strategies to avoid mastectomy skin-flap necrosis during nipple-sparing mastectomy. Br. J. Surg..

[50] Nguyen C.L., Zhou M., Easwaralingam N., Seah J.L., Chan B., Graham S., Azimi F., Mak C., Pulitano C., Warrier S. (2026). Mastectomy Skin Flap Necrosis after Implant-Based Breast Reconstruction: Intraoperative Predictors and Indocyanine Green Angiography. Plast. Reconstr. Surg..

[51] Lauritzen E., Damsgaard T.E. (2021). Use of Indocyanine Green Angiography decreases the risk of complications in autologous- and implant-based breast reconstruction: A systematic review and meta-analysis. J. Plast. Reconstr. Aesthetetic Surg..

[52] Tondu T., Hubens G., Tjalma W.A., Thiessen F.E., Vrints I., Van Thielen J., Verhoeven V. (2020). Breast reconstruction after nipple-sparing mastectomy in the large and/or ptotic breast: A systematic review of indications, techniques, and outcomes. J. Plast. Reconstr. Aesthetetic Surg..

[53] Diep G.K., Hui J.Y., Marmor S., Cunningham B.L., Choudry U., Portschy P.R., Tuttle T.M. (2016). Postmastectomy Reconstruction Outcomes After Intraoperative Evaluation with Indocyanine Green Angiography Versus Clinical Assessment. Ann. Surg. Oncol..

[54] Schols R.M., Dip F., Lo Menzo E., Haddock N.T., Landin L., Lee B.T., Malagón P., Masia J., Mathes D.W., Nahabedian M.Y. (2022). Delphi survey of intercontinental experts to identify areas of consensus on the use of indocyanine green angiography for tissue perfusion assessment during plastic and reconstructive surgery. Surgery.

[55] Pruimboom T., Schols R.M., Van Kuijk S.M., Van der Hulst R.R., Qiu S.S. (2020). Indocyanine green angiography for preventing postoperative mastectomy skin flap necrosis in immediate breast reconstruction. Cochrane Database Syst. Rev..

[56] Duggal C.S., Madni T., Losken A. (2014). An outcome analysis of intraoperative angiography for postmastectomy breast reconstruction. Aesthetetic Surg. J..

[57] Chattha A., Bucknor A., Chen A.D., Lee B.T., Lin S.J. (2018). Indocyanine Green Angiography Use in Breast Reconstruction: A National Analysis of Outcomes and Cost in 110,320 Patients. Plast. Reconstr. Surg..

[58] Lauritzen E., Bredgaard R., Laustsen-Kiel C.M., Hansen L., Tvedskov T., Damsgaard T.E. (2023). Indocyanine green angiography in oncoplastic breast surgery, a prospective study. J. Plast. Reconstr. Aesthetetic Surg..

[59] Lauritzen E.B.R., Bonde C., Jensen L.T., Tvedskov T., Damsgaard T.E. (2024). Indocyanine green angiography for autologous breast reconstruction: A prospective. Ann. Breast Surg..

[60] Mastroianni M., Lin A.M., Smith B.L., Austen W.G., Colwell A.S. (2016). Nipple Loss following Nipple-Sparing Mastectomy. Plast. Reconstr. Surg..

[61] Tigaran A.E., Avino A., Abu-Baker A., Timofan T., Ion D.E., Gheoca-Mutu D.E., Jecan R.C., Nestianu E.G., Raducu L. (2026). First Clinical Application and Validation of the Romanian BREAST-Q in Immediate and Delayed Breast Reconstruction: A Prospective Study. Cancers.

[62] Malagon-Lopez P., Vila J., Carrasco-Lopez C., Garcia-Senosiain O., Priego D., Julian Ibanez J.F., Higueras-Suñe C. (2019). Intraoperative Indocyanine Green Angiography for Fat Necrosis Reduction in the Deep Inferior Epigastric Perforator (DIEP) Flap. Aesthetetic Surg. J..

[63] Varela R., Casado-Sanchez C., Zarbakhsh S., Diez J., Hernandez-Godoy J., Landin L. (2020). Outcomes of DIEP Flap and Fluorescent Angiography: A Randomized Controlled Clinical Trial. Plast. Reconstr. Surg..

